# A prospective cohort study on the association between waterpipe tobacco smoking and gastric cancer mortality in Northern Vietnam

**DOI:** 10.1186/s12885-022-09894-6

**Published:** 2022-07-21

**Authors:** Hung Xuan Le, Dung Thi Thuy Truong, Long Bao Tran, Phuoc Hong Le, Binh Uyen Duong Pham, Koji Wada, Shunya Ikeda, Ariuntuul Garidkhuu, Can Van Phan, Ngoan Tran Le

**Affiliations:** 1 Department of Research Methodology and Biostatistics, Hanoi Medical University, Ha Noi City, Vietnam; 2 Graduate School of Public Health, International University of Health and Welfare, Narita City, Japan; 3 University of Medicine and Pharmacy at Ho Chi Minh City, Ho Chi Minh City, Vietnam; 4 Department of General Surgery, Hanoi Medical University, Ha Noi City, Vietnam; 5 School of Medicine, International University of Health and Welfare, Narita City, Japan; 6 Mongolian National University of Medical Sciences, Ulaanbaatar City, Mongolia; 7 Center for Population Health Sciences, Hanoi University of Public Health , Ha Noi City, Japan; 8 Institute of Research and Development, Duy Tan University, Da Nang City, Vietnam; 9 Department of Public Health, International University of Health and Welfare, Narita City, Japan

**Keywords:** Waterpipe tobacco, Smoking, Gastric cancer mortality, Cigarettes, Cohort, Vietnam

## Abstract

Tobacco consumption, as a worldwide problem, is a risk factor for several types of cancer. In Vietnam, tobacco consumption in the form of waterpipe tobacco smoking is common. This prospective cohort study aimed to study the association between waterpipe tobacco smoking and gastric cancer mortality in Northern Vietnam. A total of 25,619 eligible participants were followed up between 2008 and 2019. Waterpipe tobacco and cigarette smoking data were collected; semi-quantitative food frequency and lifestyle questionnaires were also utilized. Gastric cancer mortality was determined via medical records available at the state health facilities. A Cox proportional hazards model was used to estimate hazard ratios (HR) and 95% confidence intervals (95% CI). During 314,992.8 person-years of follow-up, 55 men and 25 women deaths due to gastric cancer were identified. With never-smokers as the reference, the risk of gastric cancer mortality was significantly increased in participants who were ever-smoking (HR = 2.43, 95% CI = 1.35–4.36). The positive risk was also observed in men but was not significantly increased in women. By types of tobacco use, exclusive waterpipe smokers showed a significantly increased risk of gastric cancer mortality (HR = 3.22, 95% CI = 1.67–6.21) but that was not significantly increased in exclusive cigarette smokers (HR = 1.90, 95% CI = 0.88–4.07). There was a significant positive association between tobacco smoking and gastric cancer death for indicators of longer smoking duration, higher frequency per day, and cumulative frequency of both waterpipe and cigarette smoking. Waterpipe tobacco smoking would significantly increase the risk of gastric cancer mortality in the Vietnamese population. Further studies are required to understand the waterpipe tobacco smoking-driven gastric cancer burden and promote necessary interventions.

**Supplementary Information:**

The online version contains supplementary material available at 10.1186/s12885-022-09894-6.

## Background

More than eight million people die due to tobacco use every year, and approximately 80% of tobacco consumers are from low- and middle-income countries [1]. Gastric cancer is one of the 12 cancer types associated with smoking [2]. Importantly, it is the fifth most common type of cancer and the fourth leading cause of death globally [3]. Gastric cancer is a multifactorial disease with both environmental and genetic causative factors [4]. Although chronic *Helicobacter pylori* (*H. pylori)* infection is a well-known risk factor for gastric cancer [5, 6], it is not a sufficient cause for the development of this disease [7]. Dietary and lifestyle habits are also associated with gastric cancer [8]. Tobacco smoking is directly associated with an increased risk of gastric cancer [9–11] as tobacco products often contain nitrosamine forms of chemical and many other established carcinogens that are well-known etiological agents for gastric cancer [12].

Given that tobacco consumption is associated with gastric cancer, its increased incidence and related mortality are expected to be high in countries with prominent tobacco consumption [13]. Accordingly, a previous prospective study showed a positive association between cigarette smoking and gastric cancer-related mortality [13]. However, some studies did not observe a dose–response relationship between smoking duration or intensity and gastric cancer incidence and mortality [14, 15]. Most published studies, especially prospective studies, also have limited data on the association between consumption of tobacco products (e.g., cigarettes and waterpipe tobacco) and gastric cancer [11, 14, 16, 17].

Gastric cancer is highly prevalent in Asia, with over 70% of all gastric cancer cases globally being in Asia [18]. Although Vietnam is not among the Asian countries with the highest incidence of gastric cancer, it has the lowest survival rate (35.7%) [3, 19]. Smoking is highly common in Vietnam, with a consumption rate of 22.5% (45.3% and 1.1% among men and women, respectively) [20]. Further, smoking is the second most common factor for gastric cancer cases (13.5%; 23.9% and 0.8% in men and women, respectively) [21]. Therefore, the high gastric cancer mortality might be due to the high tobacco consumption rates in Vietnam, but there is no evidence to support this.

**Fig. 1:**
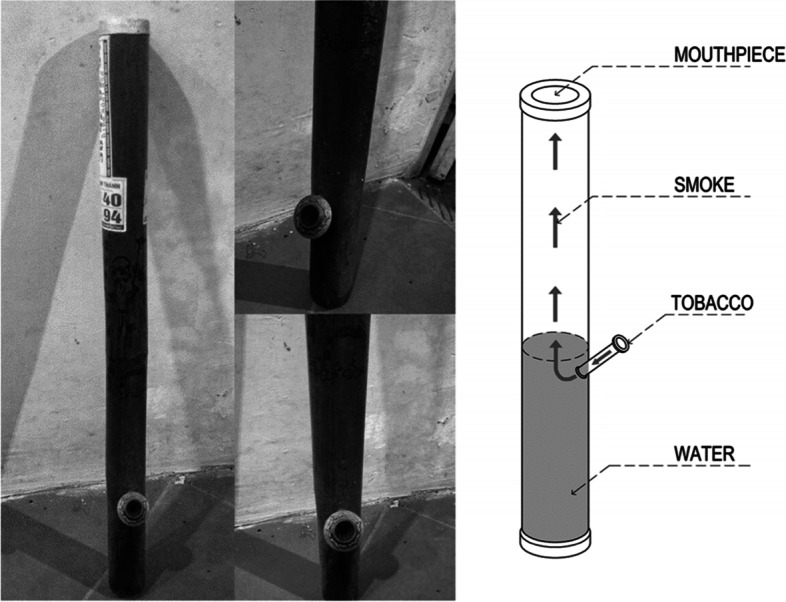
Waterpipe tobacco of Vietnamese/Chinese [10], (Reproduced with permission)

Aside from cigarettes, waterpipe tobacco smoking is also prevalent in suburban areas in Vietnam. Waterpipe tobacco is made of bamboo and is similar to Chinese bong waterpipe smoking, Fig. 1 [10].Waterpipe tobacco involves *Nicotiana rustica* leaves that contain a higher level of nicotine (9%) than cigarettes (1%–3%) [22] but is mistakenly perceived safer than cigarettes due to its water-based filter [22]. Waterpipe tobacco smoke contains higher levels of carbon monoxide (CO) and polyaromatic hydrocarbons than cigarettes; these were found to be related to lung and esophageal cancer [23]. Although waterpipe tobacco smoking indicates exposure to cancer-causing substances, large-scale quantitative evidence of the association between waterpipe tobacco smoking and gastric cancer mortality in the Vietnamese population is rare. Thus, this study aimed to investigate the association between gastric cancer mortality and waterpipe tobacco smoking in the Vietnamese population after adjusting for multiple confounding factors. Furthermore, the dose–response relationship between waterpipe tobacco smoking duration/intensity and gastric cancer mortality was explored.

## Methods

### Study design and population

**Fig. 2:**
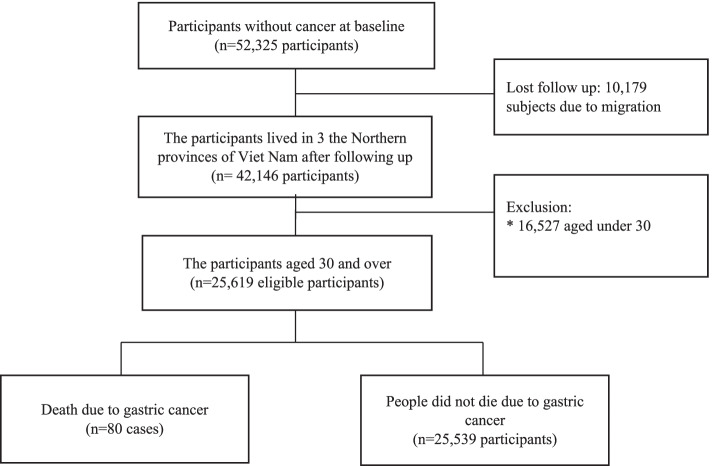
The recruitment process of study participants

This prospective cohort study involved 52,325 individuals from 12,746 households surveyed in 2008 and belonged to nine communes in three Northern provinces, including Hung Yen province, Phu Pho province, and Hanoi in Northern Vietnam. The participants were assessed in a baseline survey that included a questionnaire on waterpipe tobacco and cigarettes smoking, demographic characteristics, dietary intake, refrigerator use, cooking methods, and alcohol habits. Participants in the cohort were followed up for all causes of death, including cancer events using the medical records available at the state health facilities [24]. Cancer-related deaths were identified, and the ICD-10 code from C00-C96. The inclusion criteria were (1) no history of any cancer and (2) presence at the investigation site during the study period. After over 12 years of follow-up, 10,179 (19.5%) individuals were excluded due to migration. Another 16,527 (31.6%) participants aged < 30 years were also excluded because gastric cancer mortality rarely occurs among young people in Vietnam [17]. Finally, the data of 25,619 participants with gastric cancer mortality (*n* = 80) and no deaths due to gastric cancer (*n* = 25,539) were examined in the present study (**Fig. ****2**).

### Exposure assessment

We assessed tobacco consumption in the participants who completely smoked at least one waterpipe tobacco or cigarette during their lifetime, using a structured questionnaire and involved face-to-face interviews by the trained medical students of Hanoi Medical University.

Tobacco smoking status was classified as follows: (1) never-smokers: never-smoked cigarette/waterpipe in their lifetime; (2) former smokers: smoked cigarettes/waterpipe but had quit smoking at the time of the interview; and (3) current smokers: smoked cigarettes/waterpipe at the time of the interview.

Data on the frequency and average duration of smoking and the age at which the smoking started were collected. The exposure factors of smoking, including the age at smoking initiation, number of cigarettes smoked per day, cumulative smoking frequency, and duration of smoking, were categorized based on the distribution of the study participants. The smoking initiation age groups were divided into never, 15 − 25, and 26 − 42 years. For people without smoke daily but occasionally smoke during the week, the daily number of cigarettes smoked was calculated as the total number of cigarettes smoked during the week divided by seven days. The number of cigarettes smoked per day (frequency) was categorized into three levels overall smoking (never, one per week − 10 per day, and 11 − 100 per day), waterpipe tobacco smoking (never, one per week − 10 per day, and 11 − 75 per day), and cigarette smoking (never, one per week − 6 per day, and 7 − 80 per day). The cumulative smoking frequency was estimated by multiplying the average daily frequency of smoking (365 days) and duration of smoking (years). This index was divided into three levels for overall smoking (never, 1–164, and 165–3,250 times), waterpipe tobacco smoking (never, one − 150 times, and 156 − 3,250 times), and cigarette (never, one − 100 times, and 104 − 2,800 times). Duration of smoking (in years) was categorized into three levels overall smoking (never, 1 − 15, and 16 − 65), waterpipe tobacco smoking (never, 1 − 20, and 21 − 70), and cigarette smoking (never, 1 − 15, and 16 − 65).

### Outcome determination

All death cases, including those related to gastric cancer, were reported by each family during the first month to the state commune health station (CHS) of each commune in Vietnam. The head of the CHS reported the underlying cause of death and code following the ICD-10 [25]. Gastric cancer mortality (C16) and other causes of death were determined based on medical records available at the CHS, district hospital, provincial hospital, and other health facilities, or death certificates issued by the hospital where the patients had died [26]. Only some cases had not been admitted to any hospital and died at home; the cause of death was determined by using WHO verbal autopsies that have been validated in Vietnam. The sensitivity and positive predictive value of verbal autopsies were assessed with scores from 75 to 100% in the Vietnamese population [27]. A standard verbal autopsy instrument paired with easy-to-implement and effective analytic methods can help bridge significant gaps in information about causes of death, particularly in resource-poor settings, including Vietnam [24]. Staff involved in the study were trained and masked to baseline information coded the outcomes according to ICD-10 [25].

### Follow-up and censor of study participants

The last follow-up was completed on December 31, 2019, and the person-year of each study participant was estimated. Follow-up time in person-years was used as the underlying time and was calculated from baseline to the date of death of all causes, including gastric cancer, the date moved out of other areas, or the end of the follow-up period (December 31, 2019), whichever occurred first. The total estimated number of person-year was 314,992.8 in the present study.

### Covariate information

Potential confounding factors were selected based on previous studies that suggested an association between risk factors and gastric cancer [9]. Covariates included age; sex; education level; refrigerator availability at their household; body mass index (BMI); alcohol consumption; and total energy (Kcal/day), protein (g/day), lipid (g/day), and carbohydrate (g/day) intake. Age (years) was categorized as follows: (30–39, 40–49, 50–59, 60–69, 70–79, 80 +). The educational level was divided into < 6 years (primary school or lower level) and ≥ 6 years (secondary school and over). BMI was classified based on the recommendation of the World Health Organization for Asian populations as underweight (< 18.5 kg/m^2^), normal weight (18.5– < 23.0 kg/m^2^), and overweight (≥ 23 kg/m^2^) [28].

Refrigerator availability was either a “yes” or “no” because it represented the study participants' economic status. Alcohol consumption was divided into two groups as follows: “yes” (participants who were involved in drinking beer, wine, or hard liquor) and “no” (participants who never consumed alcohol). Dietary information was obtained using a validated semi-quantitative food frequency questionnaire (SQFFQ) to adjust for the overall diet quality. The validity of the SQFFQ was reported based on a survey of 1,334 individuals from the general population of Northern Vietnam in 2017 [29]. Regarding the SQFFQ, participants were asked to recall the frequency of consumption of each food item (using a specific portion size) over the last 12 months. Participants were given nine categories of intake to choose from: never or < 1/month, 1–3/month, 1/week, 2–4/week, 5–6/week, 1/day, 2–3/day, 4–5/day, and ≥ 6/day. Nutrient intake was calculated by multiplying the nutrient content of foods by the reported frequency of intake per year of each food from the SQFFQ and the average amount intake per day [30].

### Statistical analysis

The association between waterpipe, cigarette tobacco smoking, and gastric cancer mortality was assessed using Cox proportional hazards regression analysis to estimate hazard ratios (HR) with corresponding 95% confidence intervals (95%CI). The lifetime never-smoker group was considered the reference group in statistical analyses. Analyses were performed using the Stata software, version 10.0. All tests were two-sided, and a *p*-value of < 0.05 was considered statistically significant. The trend test was performed to evaluate the dose–response effect in multivariable-adjusted models.

## Results

### Demographic factors

During 314,992.8 person-years of follow-up, 80 gastric cancer mortalities were identified. The findings indicated that men (55 cases) had higher gastric cancer mortality than women (25 cases).

At baseline, the percentage of study participants who smoked both waterpipe and cigarette, exclusive waterpipe, and an exclusive cigarette was 9.21% (2,359/25,619), 10.75% (2,754/25,619), and 10.71% (2,745/25,619), respectively. There were 277 participants (1.08%) missing information on their smoking status. Smokers were primarily men and above 40 years old; they showed more likelihood to have higher education and alcohol consumption levels. More than 50% of study participants showed a BMI of 18.5– < 23.0 kg/m^2^, and half of them reported non-usage of the refrigerator. For alcohol usage, the estimated proportion of drinkers among ever-smokers (70.1%-80.7%) was higher than that among never-smokers (12.1%), Table 1.

**Table 1:** Characteristics of participants in the study according to tobacco smoking status in Northern Vietnam, 25,619 participants for over 12 years-following-up, 2008–2019

					**Ever smokers**					
	**Total participants (*n* = 25,619)**		**Never smoker (*n* = 17,484)**		**Both waterpipe and cigarette (*n* = 2,359)**		**Exclusive waterpipe (*n* = 2,754)**		**Exclusive Cigarette (*n* = 2,745)**	
	**Number**	**%**	**Number**	**%**	**Number**	**%**	**Number**	**%**	**Number**	**%**
**Sex**										
Men	12,144	47.4	4,369	25.0	2,263	95.9	2,631	95.5	2,619	95.4
Women	13,475	52.6	13,115	75.0	96	4.1	123	4.5	126	4.6
Total	25,619	100.0	17,484	100.0	2,359	100.0	2,754	100.0	2,745	100.0
**Age group**										
30–39	7,038	27.5	4,977	28.5	640	27.1	574	20.8	771	28.1
40–49	6,885	26.9	4,346	24.9	799	33.9	885	32.1	777	28.3
50–59	5,000	19.5	3,247	18.6	480	20.4	643	23.4	572	20.9
60–69	2,814	11.0	1,926	11.0	226	9.6	295	10.7	336	12.2
70–79	2,558	10.0	1,875	10.7	170	7.1	266	9.7	223	8.1
80 +	1,324	5.1	1,113	6.3	44	1.9	91	3.3	66	2.4
Total	25,619	100.0	17,484	100.0	2,359	100.0	2,754	100.0	2,745	100.0
**Education**										
< 6 years	6,081	23.7	4,671	26.7	400	17.0	674	24.5	289	10.5
≥ 6 years	19,276	75.3	12,589	72.0	1,955	82.9	2,059	74.8	2,446	89.1
Unknown	262	1.0	224	1.3	4	0.1	21	0.7	10	0.4
Total	25,619	100.0	17,484	100.0	2,359	100.0	2,754	100.0	2,745	100.0
**BMI (kg/m**^**2**^**)**										
< 18.5	4,844	18.9	3,423	19.6	404	17.1	542	19.7	420	15.3
18.5- < 23.0	13,944	54.5	9,455	54.1	1,305	55.3	1,516	55.1	1,512	55.1
≥ 23	2,596	10.1	1,591	9.1	304	12.9	223	8.1	449	16.4
Unknown	4,235	16.5	3,015	17.2	346	14.7	473	17.1	364	13.2
Total	25,619	100.0	17,484	100.0	2,359	100.0	2,754	100.0	2,745	100.0
**Available fridge**										
Yes	12,548	49.0	8,583	49.1	1,087	46.1	1,097	39.8	1,640	59.8
No	12,877	50.2	8,765	50.1	1,256	53.2	1,635	59.4	1,085	39.5
Unknown	194	0.8	136	0.8	16	0.7	22	0.8	20	0.7
Total	25,619	100.0	17,484	100.0	2,359	100.0	2,754	100.0	2,745	100.0
**Alcohol usage**										
Yes	7,954	31.1	2,122	12.1	1,904	80.7	1,990	72.3	1,924	70.1
No	16,654	65.0	14,608	83.6	455	19.3	764	27.7	821	29.9
Unknown	1,011	3.9	754	4.3	0	0.0	0	0.0	0	0.0
Total	25,619	100.0	17,484	100.0	2,359	100.0	2,754	100.0	2,745	100.0

*BMI* Body mass index (kg/m^2^): (Weight-kg) / (high-meter)^2^

### Waterpipe and cigarette smoking and gastric cancer mortality

Overall, we found a significantly higher gastric cancer mortality among ever-smokers participants than never-smokers (Adjusted HR = 2.43, 95% CI = 1.35 − 4.36), *p* = 0.003.

Furthermore, participants who selected the status of former cigarette, current cigarette, former waterpipe, and current waterpipe tobacco smokers showed a higher risk of gastric cancer mortality than never-smokers. However, only the current cigarette and waterpipe tobacco smoking groups displayed significant gastric cancer mortality after adjusting for potential confounding (*p* = 0.020 and *p* < 0.001, respectively). Compared with never-smokers, exclusive waterpipe smokers showed the highest risk of gastric cancer mortality (HR = 3.22, 95% CI = 1.67 − 6.21) *p* < 0.001, followed by smokers who used both waterpipe and cigarette (HR = 1.99, 95% CI = 0.89 − 4.63), *p* = 0.095, and exclusive cigarette smokers (HR = 1.90, 95% CI = 0.88 − 4.07) *p* = 0.100, Table 2. The elevated risk was also observed in both men (Adjusted HR = 2.45, 95% CI = 1.28 − 4.67), *p* = 0.007 and women (Adjusted HR = 2.21, 95% CI = 0.52 − 9.48), *p* = 0.284, Supplementary Table 1.

**Table 2:** Overall smoking status, types of tobacco, and the risk of gastric cancer mortality in Northern Vietnam among 25,619 participants for over 12 years-following-up, 2008–2019

Variables	Person-years	Case (*n* = 80)	Crude HR (95%CI)	*p* value	Adjusted HR (95%CI) ^a^	*p* value
**Overall smoking status**						
Never smoker	215,223.6	35	1.00		1.00	
Ever smoker	99,769.2	45	2.80 (1.80–4.36)	< 0.001	2.43 (1.35–4.36)	0.003
**Smoking status at baseline**						
Never smoker	215,223.6	35	1.00		1.00	
Former cigarette	20,270.7	7	2.15 (0.95–4.83)	0.065	1.35 (0.55–3.32)	0.510
Current cigarette	44,406.9	15	2.13 (1.17–3.91)	0.014	2.36 (1.14–4.87)	0.020
Former waterpipe	4,475.5	3	3.99 (1.23–12.97)	0.021	2.10 (0.61–7.23)	0.241
Current waterpipe	30,616.2	20	4.00 (2.31–6.93)	< 0.001	3.62 (1.85–7.07)	< 0.001
**By types of tobacco (missing 1 case)**						
Never smoker	215,223.6	35	1.00		1.00	
Mixed smoking of both waterpipe & cigarette	28,948.3	10	2.12 (1.05–4.28)	0.036	1.99 (0.89–4.63)	0.095
Exclusive waterpipe	33,932.7	22	3.96 (2.32–6.74)	< 0.001	3.22 (1.67–6.21)	< 0.001
Exclusive cigarette	33,458.6	12	2.30 (1.19–4.43)	0.013	1.90 (0.88–4.07)	0.100

*Abbreviation*: *HR* (95%CI) Hazard Ratio (95% confidence interval); ^a^ HR (95% CI): djusted for age groups (30–39, 40–49, 50–59, 60–69, 70–79, 80 +), sex, education level (< 6 years, ≥ 6 years), available fridge (yes/no), BMI (kg/m^2^, < 18.5, 18.5- < 23, ≥ 23), alcohol consumption (yes/no), total energy intake (Kcal/day, quintiles), protein intake (g/day, quintiles), lipid intake (g/day, quintiles), carbohydrate intake (g/day, quintiles)

There was a significant positive trend between the age at the start of smoking and gastric cancer mortality, suggesting that the start of smoking at a younger age was associated with higher gastric cancer mortality (Adjusted HR = 2.71, 95% CI = 1.27 − 5.78), *p* for trend = 0.003. Furthermore, compared with never-smokers, those who smoked tobacco 11–100 per day showed higher gastric cancer mortality (Adjusted HR = 2.31, 95% CI = 1.16 − 4.61), *p* for trend = 0.014, Table 3. A similar observation was seen in men, Supplementary Table 2. Due to the small number of cases in women, data were not examined.

**Table 3:** Mixed smoking of both waterpipe and cigarettes and the risk of gastric cancer mortality in Northern Vietnam among 25,619 participants for over 12 years-following-up, 2008–2019

Age at starting smoking (years, missing 11 cases)	Person-years	Case (*n* = 80)	Crude HR (95%CI)	*p* for trend	Adjusted HR (95%CI) ^a^	*p* for trend
Never smoker	215,223.6	35	1.00		1.00	
26–42	38,618.8	18	2.88 (1.63–5.10)		2.88 (1.38–5.99)	
15–25	44,017.9	16	2.27 (1.26–4.11)	< 0.001	2.71 (1.27–5.78)	0.003
**Frequency (session per day, missing 2 case)**						
Never smoker	215,223.6	35	1.00		1.00	
One per week-10 per day	52,706.7	25	2.98 (1.78–4.98)		2.54 (1.33–4.84)	
11–100 per day	42,203.1	18	2.62 (1.48–4.62)	< 0.001	2.31 (1.16–4.61)	0.014
**Duration of smoking (years, missing 23 cases)**						
Never smoker	215,223.6	35	1.00		1.00	
1–15	30,682.6	8	1.64 (0.76–3.55)		2.18 (0.90–5.29)	
16–65	28,977.9	14	3.01 (1.62–5.59)	< 0.001	2.35 (1.10–5.04)	0.025
**Cumulative smoking frequency (times, missing 3 cases)**						
Never smoker	215,223.6	35	1.00		1.00	
1–164	45,557.0	17	2.34 (1.31–4.18)		2.47 (1.22–4.99)	
165–3,250	46,037.7	25	3.34 (2.00–5.59)	< 0.001	2.60 (1.36–4.96)	0.004

*Abbreviation*: *HR* (95%CI) Hazard Ratio (95% confidence interval); ^a^ HR (95% CI): adjusted for age groups (30–39, 40–49, 50–59, 60–69, 70–79, 80 +), sex, education level (< 6 years, ≥ 6 years), available fridge (yes/no), BMI (kg/m^2^, < 18.5, 18.5- < 23, ≥ 23), alcohol consumption (yes/no), total energy intake (Kcal/day, quintiles), protein intake (g/day, quintiles), lipid intake (g/day, quintiles), carbohydrate intake (g/day, quintiles)

For the groups of the waterpipe plus some occasions to smoke a cigarette per week, individuals who started waterpipe tobacco smoking at 15 − 25 years of age showed an increased trend in gastric cancer mortality (Adjusted HR = 2.81, 95% CI = 1.24 − 6.36). Similarly, a longer duration (21 − 70 years), (Adjusted HR = 3.04, 95% CI = 1.51 − 6.09) and a higher frequency (11 − 75 sessions per day) of waterpipe tobacco smoking resulted in higher gastric cancer mortality (Adjusted HR = 1.87, 95% CI = 0.79 − 4.41). Furthermore, gastric cancer mortality increased with the cumulative amount of 156–3,250 waterpipe tobacco smoking (Adjusted HR = 2.69, 95% CI = 1.36 − 5.33), Table 4. In men, the start of smoking at a younger age, a longer duration, a higher frequency of smoking per day, and the cumulative amount of 156–3,250 waterpipe tobacco smoking have significantly increased the gastric cancer mortality risk, Supplementary Table 3.

**Table 4:** Waterpipe plus some occasions to smoke a cigarette and the risk of gastric cancer mortality in Northern Vietnam among 22,502 participants for over 12 years-following-up, 2008–2019

Waterpipe plus some occasions to smoke a cigarette per week	Person-years	Case (*n* = 67)	Crude HR (95%CI)	*p* for trend	Adjusted HR (95%CI) ^a^	*p* for trend
**Age at starting smoking (years, missing 6 cases)**						
Never smoker	215,223.6	35	1.00		1.00	
26–42	26,559.5	15	3.45 (1.88–6.32)		3.49 (1.64–7.44)	
15–25	27,559.8	11	2.45 (1.24–4.83)	< 0.001	2.81 (1.24–6.36)	0.001
**Frequency (session per day)**						
Never smoker	215,223.6	35	1.00		1.00	
One per week-10 per day	40,835.7	24	3.63 (2.16–6.10)		2.96 (1.55–5.62)	
11–75 per day	20,866.4	8	2.30 (1.07–4.97)	< 0.001	1.87 (0.79–4.41)	0.029
**Duration of smoking (years, missing one case)**						
Never smoker	215,223.6	35	1.00		1.00	
1–20	41,727.6	14	2.06 (1.11–3.83)		2.20 (1.05–4.62)	
21–70	18,526.2	17	5.55 (3.11–9.90)	< 0.001	3.04 (1.51–6.09)	0.001
**Cumulative frequency (times, missing one case)**						
Never smoker	215,223.6	35	1.00		1.00	
1–150	30,581.4	13	2.63 (1.39–4.97)		2.65 (1.26–5.60)	
156–3,250	28,693.2	18	3.79 (2.15–6.70)	< 0.001	2.69 (1.36–5.33)	0.004

*Abbreviation*: *HR* (95%CI) Hazard Ratio (95% confidence interval); ^a^ HR (95% CI): adjusted for age groups (30–39, 40–49, 50–59, 60–69, 70–79, 80 +), sex, education level (< 6 years, ≥ 6 years), available fridge (yes/no), BMI (kg/m^2^, < 18.5, 18.5- < 23, ≥ 23), alcohol consumption (yes/no), total energy intake (Kcal/day, quintiles), protein intake (g/day, quintiles), lipid intake (g/day, quintiles), carbohydrate intake (g/day, quintiles)

Gastric cancer mortality was also positively associated with cigarette consumption plus some occasions to smoke a waterpipe per week according to the indicators of frequency (7–80 cigarettes per day): (Adjusted HR = 2.51, 95% CI = 1.16–5.43) *p* for trend = 0.019; cumulative frequency (104–2,800): (Adjusted HR = 2.31, 95% CI = 1.03–5.17) *p* for trend = 0.032; and duration of smoking (16–65 years): (Adjusted HR = 2.38, 95% CI = 1.10–5.18) *p* for trend = 0.024 compared with the never-smoker group, Table 5. In men, a positive association between cigarette smoking and the risk of gastric cancer mortality was observed, but that is not statistically significant, Supplementary Table 4.

**Table 5:** Cigarettes plus some occasions to smoke a waterpipe and the risk of gastric cancer mortality in Northern Vietnam among 22,471 participants for over 12 years following-up, 2008–2019

Cigarettes plus some occasions to smoke a waterpipe per week	Person-years	Case (*n* = 57)	Crude HR (95%CI)	*p* for trend	Adjusted HR (95%CI) ^a^	*p* for trend
**Age at starting smoking (years, missing 7 cases)**						
Never smoker	215,223.6	35	1.00		1.00	
26–40	24,250.4	7	1.80 (0.80–4.06)		1.91 (0.74–4.95)	
15–25	26,969.3	8	1.88 (0.87–4.05)	0.047	2.34 (0.93–5.91)	0.058
**Frequency (cigarette per day, missing one case)**						
Never smoker	215,223.6	35	1.00		1.00	
One per week-6 per day	30,613.6	8	1.63 (0.76–3.53)		1.63 (0.67–3.94)	
7–80 per day	30,365.2	13	2.70 (1.43–5.11)	0.002	2.51 (1.16–5.43)	0.019
**Duration of smoking (years)**						
Never smoker	215,223.6	35	1.00		1.00	
1–15	33,150.4	9	1.71 (0.82–3.56)		2.15 (0.91–5.07)	
16–65	26,510.1	13	3.06 (1.62–5.78)	< 0.001	2.38 (1.10–5.18)	0.024
**Cumulative frequency (times, missing one case)**						
Never smoker	215,223.6	35	1.00		1.00	
1–100	33,129.1	10	1.90 (0.94–3.83)		2.19 (0.95–5.02)	
104–2,800	25,351.6	11	2.72 (1.38–5.36)	0.002	2.31 (1.03–5.17)	0.032

*Abbreviation*: *HR* (95%CI) Hazard Ratio (95% confidence interval); ^a^ HR (95% CI): adjusted for age groups (30–39, 40–49, 50–59, 60–69, 70–79, 80 +), sex, education level (< 6 years, ≥ 6 years), available fridge (yes/no), BMI (kg/m^2^, < 18.5, 18.5- < 23, ≥ 23), alcohol consumption (yes/no), total energy intake (Kcal/day, quintiles), protein intake (g/day, quintiles), lipid intake (g/day, quintiles), carbohydrate intake (g/day, quintiles)

## Discussions

This large-scale, population-based cohort study examined the association between tobacco smoking, particularly waterpipe tobacco smoking, and gastric cancer mortality in Vietnam. The results showed an elevated mortality risk among smokers and suggested differences in the impact of cigarettes and waterpipe tobacco smoking on gastric cancer mortality. Further, there was a positive dose–response relationship between waterpipe tobacco smoking and gastric cancer mortality, with a higher risk among smokers who started smoking at a younger age, consumed more waterpipe tobacco per day, and had a longer smoking duration than among never-smokers. Similarly, the significantly elevated risk of gastric cancer mortality was also confirmed in cigarette smokers.

This study revealed that gastric cancer mortality in men was higher than in women (55 cases versus 25 cases, respectively). Environmental and genetic risk factors contributed to the gastric cancer mortality rate of both men and women. *H. pylori* infection has been proven to be the major risk factor for gastric cancer. Infection of *H. pylori* was most common in men than women [31]. Smoking is also an important risk factor for gastric cancer [10, 32]. Globally, it is estimated that smoking accounts for 16.5% and 1.9% of gastric cancer-related deaths in men and women, respectively [33]. In Vietnam, a recent study reported that tobacco smoking contributes to 13.5% of all cancer incidences in Vietnam (23.9% in men and 0.8% in women) [21]. Additionally, smoking prevalence in men is higher than in women (45.3% among men and 1.1% among women) [20]. A meta-analysis had supported the hypothesis that extended exposure to estrogen effects of either ovarian or exogenous origin may decrease gastric cancer mortality [34]. The underlying reasons are unclear, but various mechanisms are being suggested. There is evidence that estrogen may lead to increased expression of trefoil factor proteins, which protect mucous epithelia or inhibit oncogene expression [35].

The current study showed that the ever-smokers status, either waterpipe tobacco or cigarettes, was associated with a higher risk of gastric cancer mortality than never-smokers. Although tobacco smoking has been proven to be an independent risk factor for gastric cancer [10, 32] evidence of its association with gastric cancer mortality remains limited. A cohort study in the United Kingdom reported a 1.63% higher mortality risk due to gastric cancer among current smokers than among non-smokers [15]. Similarly, Fujino et al. [14] also indicated that the current Japanese smokers represented an elevated risk of gastric cancer mortality.

Tobacco smoke contains numerous carcinogens that promote cancer development, including gastric cancer [32, 36]. Several possible mechanisms have been suggested to explore the impact of smoking habits on gastric cancer; it may induce peptic ulcers and chronic inflammation. Other underlying possible smoking-driven mechanisms include increased pepsin and acid secretion, gastric motility, reflux of duodenal bile salts back into the gastric space, changes in blood flow at the inflammatory sites, and alteration in mucosal cell proliferation [37]. Moreover, tobacco smoking may act together with *H. pylori* infection, a leading risk factor for gastric cancer, and markedly increase cancer risk [16, 38, 39].

Smoking is unlikely to increase the incidence of *H. pylori* infection [36]. Still, it increases the inflammatory reaction to an already established *H. pylori* infection [40] and contributes to an excess of persistent *H. pylori* infections due to the adverse effects of smoking on the immune system [41]. Several studies have also reported that the concentrations of several vitamins, which showed a protective effect on gastric cancer development, including vitamin A, vitamin C, and vitamin E, significantly decrease among smokers as compared to non-smokers [42–44]. These findings suggest that tobacco smoking considerably impacts gastric cancer development.

In this study, the effect of tobacco smoking and *H. pylori* infection on gastric cancer could not be examined due to the unavailability of *H. pylori* infection data. However, the combined impact of these two factors should be considered in further studies, particularly in Vietnam, due to the high prevalence of both tobacco smoking [20] and *H. pylori* infection [45, 46].

The current study assessed the dose–response relationships between the intensity and duration of smoking and gastric cancer mortality. The results indicated the positive association between the intensity and duration of smoking with gastric cancer for all types of tobacco smoking, including either cigarettes or waterpipe tobacco smoking. The International Agency for Research on Cancer reports that most case–control studies observed statistical associations between these two parameters, but the results from cohort studies are inconsistent [36]. Another review of 40 cohort studies supported a dose–response relationship with caution [11]. In contrast, a recent study using a dataset of 23 epidemiological reports supported the association [31]. The uncertain results may be due to the varied carcinogenic agents rather than tobacco smoking inducing gastric cancer, including *H. pylori* infection, rubber production industry, X-radiation, and gamma radiation [47]. Another reason may be the lack of separation of different types of tobacco exposures. The present study supports a dose–response relationship between tobacco smoking and gastric cancer even though data on cancer mortality was used.

The current study suggested that waterpipe tobacco smoking is likely to cause more harmful effects than cigarette smoking when a significantly greater risk of gastric cancer mortality was found in exclusive waterpipes but not for an exclusive cigarette. Similar to Chinese waterpipe smokers, Vietnamese ones use *Nicotiana rustica* leaves containing extremely high levels of nicotine and other chemicals compared with the moderate amounts found in *Nicotiana tabacum* (cigarettes) [48]. Compared with the Arabian, the Chinese waterpipe may show lower carcinogenic effects because of the non-use of charcoal and generally shorter smoking duration; however, it is associated with significantly high levels of carbon monoxide [22]. Moreover, the particulate matter 2.5 levels among waterpipe smokers are twice as high as those among cigarette smokers [22]. Long-term exposure to particulate matter 2.5 is associated with increased gastric cancer mortality [49].

In Vietnam, waterpipe tobacco smoking is the second most common type of smoking (29.8%) after cigarette (80.6%) smoking [20]. Waterpipe smokers are mistakenly perceived as safer than cigarettes; however, this study proved that waterpipe tobacco smoking is associated more with gastric cancer mortality. This is consistent with a previous case–control study on the Vietnamese population [10]. Although an updated report of the World Health Organization study group on tobacco product regulation in 2019 recommends suspending *Nicotiana rustica* [48], further analysis is required to examine the different impacts of Chinese waterpipes.

To the best of our knowledge, this is the first large cohort study to analyze the impact of tobacco smoking on gastric cancer mortality in Vietnam. However, this study also has some limitations. First, the small number of gastric cancer mortality cases limited our analysis to classify participants into exclusive waterpipe or cigarette groups, which may lead to an underestimation of the impact of waterpipe on gastric cancer death. Second, using mortality as an endpoint for the analysis may change the relationship between smoking and gastric cancer; the impact of smoking may seem reduced. Third, the interaction between tobacco smoking and *H. pylori* infection could not be examined due to the lack of information on *H. pylori* infection. Lastly, some participants were lost to follow-up because of their migration, which might have influenced the present findings. In practice, it is difficult to follow up on the participants’ migration to a developing country with limited resources. These limitations need to be addressed in future studies. 

## Conclusions

Waterpipe and cigarette tobacco smoking were associated with increased gastric cancer mortality in a large Vietnam prospective cohort dataset. The dose–response relationship between waterpipe tobacco smoking and gastric cancer mortality was found even though it still warrants further investigation to make a definitive conclusion.

## Supplementary Information

**Additional file 1.**

## Data Availability

The datasets analyzed during the current study are not publicly available due to participant privacy restrictions but are available from the corresponding author on reasonable request.

## Abbreviations

BMI: Body mass index
CHS: Commune health station
CI: Confidence intervals
HR: Hazard ratio
H. pylori: Helicobacter pylori
SQFFQ: A semi-quantitative food frequency questionnaire
