# Supplementary material for: A prospective cohort study on the association between waterpipe tobacco smoking and gastric cancer mortality in Northern Vietnam
Source: BMC Cancer. 2022 Jul 21;22:803. doi: 10.1186/s12885-022-09894-6 (PMC9306202; doi:10.1186/s12885-022-09894-6)
Supplement: Supplementary file 1 — Additional file 1. [file 12885_2022_9894_MOESM1_ESM.docx]

**Supplementary 1**

Table 1: Overall smoking status, types of tobacco, and the risk of stomach cancer by sex in the Northern Vietnam among 25,619 participants for over 12 years-following-up, 2008-2019

| **Variables** | **Men** | | | | **Women** | | | |
| --- | --- | --- | --- | --- | --- | --- | --- | --- |
|  | **Case**  **(n= 55)** | **Adjusted HR^a^ (95%CI)** | **p value** | **Case**  **(n= 25)** | | **Adjusted HR^a^ (95%CI)** | **p value** |  |
| **Overall Smoking status** |  |  |  |  | |  |  |  |
| Never smoker | 12 | 1.00 (Reference) |  | 23 | | 1.00 (Reference) |  |  |
| Ever smoker | 43 | 2.45 (1.28-4.67) | 0.007 | 2 | | 2.21 (0.52-9.48) | 0.284 |  |
| **Smoking status at baseline** |  |  |  |  | |  |  |  |
| Never smoker | 12 | 1.00 (Reference) |  | 23 | | // | // |  |
| Former cigarette | 6 | 1.27 (0.47-3.38) | 0.637 | 1 | | // | // |  |
| Current cigarette | 14 | 2.27 (1.02-5.02) | 0.043 | 1 | | // | // |  |
| Former waterpipe | 3 | 2.39 (0.67-8.51) | 0.179 | 0 | | // | // |  |
| Current waterpipe | 20 | 3.81 (1.84-7.87) | <0.001 | 0 | | // | // |  |
| **By types of tobacco (missing one case in men)** |  |  |  |  | |  |  |  |
| Never smoker | 12 | 1.00 (Reference) |  | 23 | | // | // |  |
| Mixed smoking both waterpipe & cigarette | 10 | 2.16 (0.91-5.14) | 0.081 | 0 | | // | // |  |
| Waterpipe only | 22 | 3.56 (1.72-7.36) | 0.001 | 0 | | // | // |  |
| Cigarette only | 10 | 1.72 (0.73-4.07) | 0.216 | 2 | | // | // |  |

Abbreviation: HR (95%CI), Hazard Ratio (95% confidence interval); ^a^ HR (95% CI): adjusted for age groups (30-39, 40-49, 50-59, 60-69, 70-79, 80+), sex, education level (< 6 years, ≥ 6 years), available fridge (yes/no), BMI (kg/m^2^, <18.5, 18.5-<23, ≥23), alcohol consumption (yes/no), total energy intake (Kcal/day, quintiles), protein intake (g/day, quintiles), lipid intake (g/day, quintiles), carbohydrate intake (g/day, quintiles).

//: cannot calculate due to the small number of participants.

Table 2: Mixed smoking of both waterpipe and cigarettes and the risk of gastric cancer mortality by sex in the Northern Vietnam among 25,619 participants for over 12 years-following-up, 2008-2019

| **Variables** | **Men** | | | **Women** | | |
| --- | --- | --- | --- | --- | --- | --- |
|  | **Case**  **(n= 55)** | **Adjusted HR^a^ (95%CI)** | **p for trend^b^** | **Case**  **(n= 25)** | **Adjusted HR^a^ (95%CI)** | **p for trend^b^** |
| **Age at starting smoking (years)** |  |  |  |  |  |  |
| Never smoker | 12 | 1.00 (Reference) |  | 23 | // | // |
| 26-42 | 17 | 2.86 (1.32-6.21) |  | 1 | // | // |
| 15-25 | 16 | 2.79 (1.26-6.18) | 0.004 | 0 | // | // |
| Unknown | 10 |  |  | 1 |  |  |
| **Frequency (session per day)** |  |  |  |  |  |  |
| Never smoker | 12 | 1.00 (Reference) |  | 23 | // | // |
| 1-10 | 23 | 2.57 (1.24-5.30) |  | 2 | // | // |
| 11-100 | 18 | 2.53 (1.19-5.39) | 0.016 | 0 | // | // |
| Unknown | 2 |  |  | 0 |  |  |
| **Duration of smoking (years)** |  |  |  |  |  |  |
| Never smoker | 12 | 1.00 (Reference) |  | 23 | // | // |
| 01-15 | 8 | 2.13 (0.82-5.53) |  | 0 | // | // |
| 16-65 | 12 | 2.07 (0.91-4.73) | 0.078 | 2 | // | // |
| Unknown | 23 |  |  | 0 |  |  |
| **Cumulative smoking frequency (times)** |  |  |  |  |  |  |
| Never smoker | 12 | 1.00 (Reference) |  | 23 | // | // |
| 1-164 | 17 | 2.71 (1.24-5.89) |  | 0 | // | // |
| 165-3,250 | 23 | 2.67 (1.30-5.50) | 0.009 | 2 | // | // |
| Unknown | 3 |  |  | 0 |  |  |

Abbreviation: HR (95%CI), Hazard Ratio (95% confidence interval); ^a^ HR (95% CI): adjusted for age groups (30-39, 40-49, 50-59, 60-69, 70-79, 80+), sex, education level (< 6 years, ≥ 6 years), available fridge (yes/no), BMI (kg/m^2^, <18.5, 18.5-<23, ≥23), alcohol consumption (yes/no), total energy intake (Kcal/day, quintiles), protein intake (g/day, quintiles), lipid intake (g/day, quintiles), carbohydrate intake (g/day, quintiles).

^b^ p-value for trend of those in unknow categories were excluded.

//: cannot calculate due to the small number of participants.

Table 3: Waterpipe plus some occasions to smoke a cigarette and the risk of gastric cancer mortality by sex in Northern Vietnam among 22,502 participants for over 12 years-following-up, 2008-2019

| **Variables** | **Men** | | | **Women** | | |
| --- | --- | --- | --- | --- | --- | --- |
|  | **Case**  **(n= 55)** | **Adjusted HR^a^ (95%CIs)** | **p for trend^b^** | **Case**  **(n= 25)** | **Adjusted HR^a^ (95%CIs)** | **p for trend^b^** |
| **Age at starting smoking (years)** |  |  |  |  |  |  |
| Never smoker | 12 | 1.00 (Reference) |  | 23 | // | // |
| 26-42 | 15 | 4.04 (1.81-9.03) |  | 0 | // | // |
| 15-25 | 11 | 3.16 (1.33-7.49) | 0.001 | 0 | // | // |
| Unknown | 17 |  |  | 2 |  |  |
| **Frequency (session per day)** |  |  |  |  |  |  |
| Never smoker | 12 | 1.00 (Reference) |  | 23 | // | // |
| 1-10 | 24 | 3.67 (1.78-7.56) |  | 0 | // | // |
| 11-75 | 8 | 2.32 (0.92-5.84) | 0.015 | 0 | // | // |
| Unknown | 11 |  |  | 2 |  |  |
| **Duration of smoking (years)** |  |  |  |  |  |  |
| Never smoker | 12 | 1.00 (Reference) |  | 23 | // | // |
| 01-20 | 14 | 2.63 (1.15-6.02) |  | 0 | // | // |
| 21-70 | 17 | 3.83 (1.79-8.18) | <0.001 | 0 | // | // |
| Unknown | 12 |  |  | 2 |  |  |
| **Cumulative smoking frequency (times)** |  |  |  |  |  |  |
| Never smoker | 12 | 1.00 (Reference) |  | 23 | // | // |
| 01 – 150 | 13 | 3.34 (1.45-7.68) |  | 0 | // | // |
| 156 – 3,250 | 18 | 3.37 (1.59-7.18) | 0.001 | 2 | // | // |
| Unknown | 12 |  |  | 0 |  |  |

Abbreviation: HR (95%CI), Hazard Ratio (95% confidence interval); ^a^ HR (95% CI): adjusted for age groups (30-39, 40-49, 50-59, 60-69, 70-79, 80+), sex, education level (< 6 years, ≥ 6 years), available fridge (yes/no), BMI (kg/m^2^, <18.5, 18.5-<23, ≥23), alcohol consumption (yes/no), total energy intake (Kcal/day, quintiles), protein intake (g/day, quintiles), lipid intake (g/day, quintiles), carbohydrate intake (g/day, quintiles).

^b^ p-value for trend of those in unknow categories were excluded.

//: cannot calculate due to the small number of participants.

Table 4: Cigarettes plus some occasions to smoke a waterpipe and the risk of gastric cancer mortality by sex in Northern Vietnam among 22,471 participants for over 12 years-following-up, 2008-2019

| **Variables** | **Men** | | | **Women** | | |
| --- | --- | --- | --- | --- | --- | --- |
|  | **Case**  **(n= 55)** | **Adjusted HR^a^ (95%CI)** | **p for trend^b^** | **Case**  **(n= 25)** | **Adjusted HR^a^ (95%CI)** | **p for trend^b^** |
| **Age at starting smoking (years)** |  |  |  |  |  |  |
| Never smoker | 12 | 1.00 (Reference) |  | 23 | // | // |
| 26-40 | 6 | 1.61 (0.58-4.48) |  | 1 | // | // |
| 15-25 | 8 | 2.13 (0.82-5.55) | 0.132 | 0 | // | // |
| Unknown | 29 |  |  | 1 |  |  |
| **Frequency (cigarette per day)** |  |  |  |  |  |  |
| Never smoker | 12 | 1.00 (Reference) |  | 23 | // | // |
| 01-06 | 8 | 1.63 (0.64-4.10) |  | 0 | // | // |
| 07-80 | 11 | 2.15 (0.92-5.02) | 0.075 | 2 | // | // |
| Unknown | 24 |  |  | 0 |  |  |
| **Duration of smoking (years)** |  |  |  |  |  |  |
| Never smoker | 12 | 1.00 (Reference) |  | 23 | // | // |
| 01-15 | 9 | 2.11 (0.84-5.28) |  | 0 | // | // |
| 16-65 | 11 | 2.08 (0.89-4.84) | 0.078 | 2 | // | // |
| Unknown | 23 |  |  | 0 |  |  |
| **Cumulative smoking frequency (times)** |  |  |  |  |  |  |
| Never smoker | 12 | 1.00 (Reference) |  | 23 | // | // |
| 01 – 100 | 10 | 2.18 (0.90-5.30) |  | 0 | // | // |
| 104 – 2,800 | 9 | 1.97 (0.81-4.79) | 0.108 | 2 | // | // |
| Unknown | 24 |  |  | 0 |  |  |

Abbreviation: HR (95%CI), Hazard Ratio (95% confidence interval); ^a^ HR (95% CI): adjusted for age groups (30-39, 40-49, 50-59, 60-69, 70-79, 80+), sex, education level (< 6 years, ≥ 6 years), available fridge (yes/no), BMI (kg/m^2^, <18.5, 18.5-<23, ≥23), alcohol consumption (yes/no), total energy intake (Kcal/day, quintiles), protein intake (g/day, quintiles), lipid intake (g/day, quintiles), carbohydrate intake (g/day, quintiles).

^b^ p-value for trend of those in unknow categories were excluded.

//: cannot calculate due to the small number of participants.
